# Temperature Acclimation Alters the Thermal Tolerance and Intestinal Heat Stress Response in a Tibetan Fish *Oxygymnocypris stewarti*

**DOI:** 10.3389/fmicb.2022.898145

**Published:** 2022-06-23

**Authors:** Tingbing Zhu, Xuemei Li, Xingbing Wu, Deguo Yang

**Affiliations:** Key Laboratory of Freshwater Biodiversity Conservation, Ministry of Agriculture and Rural Affairs of China, Yangtze River Fisheries Research Institute, Chinese Academy of Fishery Sciences, Wuhan, China

**Keywords:** temperature acclimation, *Oxygymnocypris stewarti*, thermal tolerance, heat stress, intestinal microbiome

## Abstract

Numerous studies have shown that thermal tolerance and intestinal heat resistance are strongly associated with temperature acclimation. However, few reports have successfully conducted similar research on fishes from the Qinghai–Tibetan Plateau, an area that is facing the threat of climate warming. Therefore, the present study determined the growth, thermal tolerance, and intestinal heat stress (exposure to 30°C) responses in juveniles of a Tibetan fish, *Oxygymnocypris stewarti*, acclimated to three temperature levels (10°C, 15°C, and 20°C, named as T10, T15, and T20, respectively) for 30 days. The fastest growth was recorded in the T15 group. At 1°C/30 min heating rate, the critical thermal maximum (*CT_Max_*) ranged from 31.3°C to 32.3°C, and the lethal thermal maximum (*LT_Max_*) ranged from 31.8°C to 32.6°C among the three acclimation temperatures. According to the results of thermal tolerance tests, the heat stress temperature was set to 30°C. When the water temperature reached 30°C, the expression of the intestinal heat shock protein 70 (*HSP70*) gene as well as the intestinal microbiome and histology of experimental fish were monitored at 0, 2, 6, and 12 h. The expression of *HSP70* reached the highest level at 2 h in all three temperature treatments. The histological analysis showed damage to intestinal cells, including diffuse infiltration of lymphocytes, villi epithelial cell swelling, decrease of intestinal villi length, and cytoplasmic light staining at 2 h in all three temperature treatments. In terms of the intestinal microbiome, phyla Proteobacteria and Firmicutes dominated the treatments at each monitored time in the T10 and T15 groups and at 0 h in T20 group, while phyla Fusobacteria, Proteobacteria, and Cyanobacteria were dominant in treatments at 2, 6, and 12 h in the T20 group. The overall results indicated that acclimation temperature could affect the growth, thermal tolerance, and intestinal heat stress response of *O. stewarti* juveniles. As the first report on intestinal heat stress response associated with temperature acclimation in a Tibetan fish, this study will help to understand the potential effects of climate change on highland fishes.

## Introduction

Temperature, a variable that is widely recognized as a crucial environmental factor for organisms, affects fish life history traits such as growth (Moltschaniwskyj and Martınez, 1998; Desai and Singh, 2009; He et al., 2014), development (Alami-Durante et al., 2000), reproduction (Leggett and Carscadden, 1978), routine metabolism (Luo and Wang, 2012), and energy allocation (Sogard and Spences, 2004). With global warming, the response of fishes from the Qinghai–Tibetan Plateau have gained widespread attention due to most of the Tibetan fishes being cold water fish and the Tibetan Plateau being highly sensitive to climate change (Liu and Chen, 2000). As the main water system in the Tibetan Plateau, the Yarlung Zangbo River basin was reported to have warmed during 1961–2005 (You et al., 2007), indicating that the Tibetan fishes are facing threats due to global warming. Thus, the response of Tibetan fishes to climate change should be significant for both researchers and management.

Thermal tolerance, the production of heat shock proteins, and histological changes from heat stress are critical to understanding the response mechanism to climate warming. The critical thermal maximum (*CT_Max_*) and the lethal thermal maximum (*LT_Max_*) are commonly used for equating an animal’s heat tolerance to the temperature at which it loses the ability to escape from constant rapid warming (Paladino et al., 1980). Many studies have shown remarkable effects of acclimation temperature on the thermal tolerance of fish, indicating the plasticity of thermal tolerance (Sarma et al., 2010; Underwood et al., 2012; He et al., 2014). Exposure to heat induces the production of heat shock protein 70 (*HSP70*) in the intestine, liver, and other tissues (Hotchkiss et al., 1993). Acute heat stress may damage animal tissues and organs (Bouchama and Knochel, 2002; Cronjé, 2005).

In recent years, intestinal microbiome variation induced by heat stress has also been increasingly studied. The intestinal microbiota influences multiple features of the host’s biology, including nutrient acquisition, immune response, metabolism, behavior, and life history traits (Broderick and Lemaitre, 2012; Douglas, 2018; Hoye and Fenton, 2018). Heat stress significantly changes intestinal microbiome diversity (Cao et al., 2021; Jaramillo and Castañeda, 2021; Wen et al., 2021; Yi et al., 2021). Meanwhile, the intestinal microbiota can contribute to host thermal tolerance (Kokou et al., 2018). The ongoing climate change is expected to impose strong selection pressure on the heat tolerance of ectotherms (Hoffmann and Sgrò, 2011). Therefore, knowledge of intestinal microbiome variation associated with heat stress is of special significance to understanding the adaptions to climate warming.

*Oxygymnocypris stewarti* is a Tibetan fish species distributed in the main body and tributaries of the middle reaches of the Yarlung Zangbo River (Bureau of Aquatic Products, Tibet, China, 1995). The maximum natural habitat temperature of *O. stewarti* never exceeded 20°C (Li et al., 2010). Due to overfishing and habitat destruction, the distribution area and population resources of *O. stewarti* are shrinking. To enhance the protection of this species, *O. stewarti* is listed as near threatened on the IUCN Red List and is classified as endangered at the national level. To date, most reports concerning *O. stewarti* have focused on age and growth (Jia and Chen, 2011; Huo et al., 2012), feeding habits (Huo et al., 2014), otolith microstructure (Jia and Chen, 2009), reproductive biology (Huo et al., 2013), embryonic and larval development (Xu et al., 2011), genetic structure (Dong et al., 2021), nutrition composition (Luo et al., 2014), and the genome (Liu et al., 2019). For the intestinal microbial communities in *O. stewarti*, Chen et al. (2017) compared the differences between healthy and diseased individuals. However, no study has examined the thermal tolerance or heat stress induced intestinal microbiome variation in *O. stewarti*.

The aims of the present study were: (1) to explore the suitable growth temperature and the thermal tolerance of juvenile *O. stewarti* at three acclimation temperatures (10°C, 15°C, and 20°C); and (2) to examine the expression of intestinal *HSP70*, the intestinal histology, and the microbiome of juvenile *O. stewarti* in response to acute heat stress (30°C) after acclimation at the three temperatures mentioned above. The results will be valuable in evaluating the effects of temperature acclimation on thermal tolerance and intestinal heat resistance of Tibetan fishes.

## Materials and Methods

### Experimental Fish and Acclimation Procedure

All of the animal experiments in the present study were approved by the Institutional Animal Care and Use Committee of Yangtze River Fisheries Research Institute, Chinese Academy of Fishery Sciences, and they were performed following the institutional ethical guidelines for experimental animals. The experimental fish were hatchery *O. stewarti* juveniles from Tibet. The parents of the experimental fish were wild mature *O. stewarti* caught from the Yarlung Zangbo River. In January 2016, about 1,000 juveniles were chosen and stocked in three conical polypropylene tanks (diameter = 80 cm, water volume = 300 L, and water flow rate = 0.286 L/s) connected to a recirculating aquaculture system. Fish were held in the water at 15°C for 2 weeks prior to the experiment, then were acclimated at three temperatures of 10°C, 15°C, and 20°C (groups T10, T15, and T20) at a rate of 2°C/day using a 1.47 KW aquarium refrigerating and heating machine (temperature control accuracy 0.1°C). For each temperature group, two tanks were used with a density of 45 individuals per tank. After the target temperature was stable (deviation <0.5°C) for 1 week, fish were initiated into the acclimation procedure for 30 days. Fish were fed with 1% of body weight artificial dry diet twice (09:00 and 15:00) per day. The dissolved oxygen was above 8 mg/L, and the photoperiod was 12 L:12D.

### Thermal Tolerance Test and Sampling

The thermal tolerance tests were conducted in three conical polypropylene tanks. All of the fish were starved for 24 h prior to the tests. For each acclimation temperature level, 30 fish (10 from each replicate) were chosen at random for the test. The values of *CT_Max_* and *LT_Max_* were determined at a heating rate 1°C/30 min. The *CT_Max_* and *LT_Max_* values were calculated as the arithmetic mean of the collective endpoint of individuals (Beitinger et al., 2000). After the test, fish were transported to the water of their acclimation temperature for recovery and the criteria of success recovery was to recover spontaneous breathing within 12 h.

### Heat Stress Test and Sampling

The heat stress tests were also conducted in another set of conical polypropylene tanks. The heat stress temperature was set to 30°C based on the results of the thermal tolerance test, which showed the experimental fish reached the most manic state at 30°C. For each acclimation temperature group (T10, T15, and T20), 30 fish were chosen and transferred into the same tank with a water temperature of 30°C. Then, three experimental fish from each tank were sampled at random after 0, 2, 6, and 12 h during the heat stress procedure; the treatments were named T10-0h, T10-2h, T10-6h, T10-12h; T15-0h, T15-2h, T15-6h, T15-12h; T20-0h, T20-2h, T20-6h, and T20-12h. Samples at 12 h were unavailable for the 10°C group, as all of the experimental fish were dead after 6 h of stress. For all of the fish samples, the intestine was first carefully removed under sterile conditions, and all of the hindgut samples except for two samples at 12 h of the 10°C and 15°C groups were separated and collected in 2-ml freezing centrifuge tubes and stored at −80°C for subsequent microbial DNA extraction. The remaining intestinal fragments were washed with saline and divided into foregut and midgut. The foregut samples were fixed in 4% neutral paraformaldehyde solution for histological analysis, while the midgut samples were quickly stored in liquid nitrogen for further detection of *HSP70* expression.

### Expression of *HSP70*

Total RNA of the intestine was extracted from each sample using a DP431 Reagent RNA kit (Tiangen, China) according to Li et al. (2015). RNA was dissolved in 50 ml RNase-free water and stored at −70°C until use. cDNA was synthesized for quantitative reverse transcription PCR (RT-qPCR) using the RevertAid First Strand cDNA Synthesis Kit (Thermo Scientific, China) according to the manufacturer’s instructions. The qPCR primers were designed using Primer 5.0 software based on the available cytokine sequences in GenBank. The qPCR was performed in a StepOnePlus™ Software real-time PCR Detection system (ABI, Beijing, China). Each 20-μl real time PCR reaction mixture consisted of 10 μl SYBR Select Master Mix (2×; TIANGEN, Beijing, China), 1 μl primer of each, 2 μl cDNA, and 6 μl distilled/deionized H_2_O. The cycling conditions were as follows: 95°C for 10 min and then 40 cycles of 95°C for 15 s, 60°C for 60 s, and 60°C for 5 min. All of the qPCRs were performed at least three times. Data analysis was conducted using high resolution melt (HRM) software.

### Intestinal Microbiome DNA Extraction, PCR, and Sequencing

The intestinal microbial DNA was extracted using the Powerfecal DNA Isolation kit (Mo Bio Laboratories Inc., Carlsbad, CA, United States) in accordance with the manufacturer’s protocols. The 515F (GTGCC AGCMGCCGCGGTAA) and 909R (CCCCGYCAATTCMTTTRAGT) primers were used to amplify the bacterial 16S rRNA gene V4–V5 fragments. PCR integrant and protocols were carried out as: 94°C for 3 min followed by 30 cycles of 94°C for 40 s, 56°C for 60 s, 72°C for 60 s, and a final extension at 72°C for 10 min until the reaction was halted by the user.

The PCR products were separated by 2% agarose gel electrophoresis, and negative controls were always performed to make sure there was no contamination. All bands of the desired size of approximately 420 bp were purified using the AxyPrep DNA Gel Extraction Kit (Axygen Biosciences, Union City, CA, United States). The bar-coded amplicons from each sample were pooled in equimolar concentrations and then were sequenced on an Illumina MiSeq platform (Guangdong Meilikang Bio-science Ltd., China) according to the standard protocols.

### Data Processing and Taxonomy Assignment

QIIME (v1.9.0) was used to process and quality-filter the raw fastq files according to a quality-control process (Caporaso et al., 2010). All the sequence reads were trimmed and assigned to each sample based on their barcodes. The sequences with high quality (length > 300 bp, without ambiguous base “N,” and average base quality score > 30) were used for downstream analysis. Chimera sequences were removed using the UCHIME algorithm (Edgar et al., 2011). The processed sequences with ≥97% similarity were clustered into the same Operational Taxonomic Units (OTUs) by the UCLUST algorithm. Taxonomic assignments of each OTU were determined using the RDP classifier (Wang et al., 2007).

### Intestinal Histology

Intestinal tissues were fixed in 4% neutral paraformaldehyde solution for 7 days, then washed, dehydrated, made transparent, waxed, embedded in paraffin, cut 4- to 5-μm-thick slices, and routinely stained with hematoxylin and eosin (HE). The tissue sections were used in light microscopy and the length of intestinal villus were measured.

### Statistical Analysis

The mean specific growth rate of body weight (SGR_W_) of each acclimation group was assessed by the following function:


$$SGR_{W}\;\left(\%/day\right)=100\times \left(\ln W_{t}-\ln W_{0}\right)/t,$$

where *W*_0_ and *W_t_* are the mean body weights of experimental fish before and after acclimation, respectively; *t* is the period of the acclimation experiment.

A correlation heatmap of dominant bacterial genera was analyzed using the corrplot R package, and Beta diversity was calculated by unconstrained principal coordinates analysis (PCoA) based on weighted UniFrac distance. One way ANOVA and multiple comparison tests were used to compare the differences in individual size and growth among the acclimation treatment groups. The data relating to the microbial community and expression of *HSP70* were analyzed on the free online Tutools Platform. A significant difference was set at a value of *p* < 0.05.

## Results

### Growth and Thermal Tolerance Under Different Acclimation Temperatures

The mean *SGR_W_* of fish in the T15 group was more rapid than those of the T10 and T20 groups (Table 1). Both *CT_Max_* and *LT_Max_* increased with increasing acclimation temperature (Table 1). The mean recovery rates of fish in the thermal tolerance test were 14.4%, 22.8%, and 23.3% for T10, T15, and T20 group, respectively.

**Table 1:** Final size, specific growth rate of body weight (*SGR_W_*), upper thermal tolerance (*CT_Max_*), and lethal thermal maximum (*LT_Max_*) of *Oxygymnocypris stewarti* juveniles in three acclimation temperature levels.

Acclimation temperature (°C)	10	15	20
Initial body weight (g)	5.42 ± 1.35^a^	4.60 ± 1.31^b^	4.82 ± 1.24^b^
Final body weight (g)	5.94 ± 1.67	5.83 ± 1.37	5.80 ± 1.36
*SGRw* (%/day)	0.31	0.79	0.62
*CT_Max_* (°C)	31.3	32.1	32.3
*LT_Max_* (°C)	31.8	32.6	32.5

Means marked with different superscripts in each column indicate a significant difference between the means (*p* < 0.05).

### Variation in Expression of Intestinal *HSP70* Under Heat Stress

**Figure 1:**
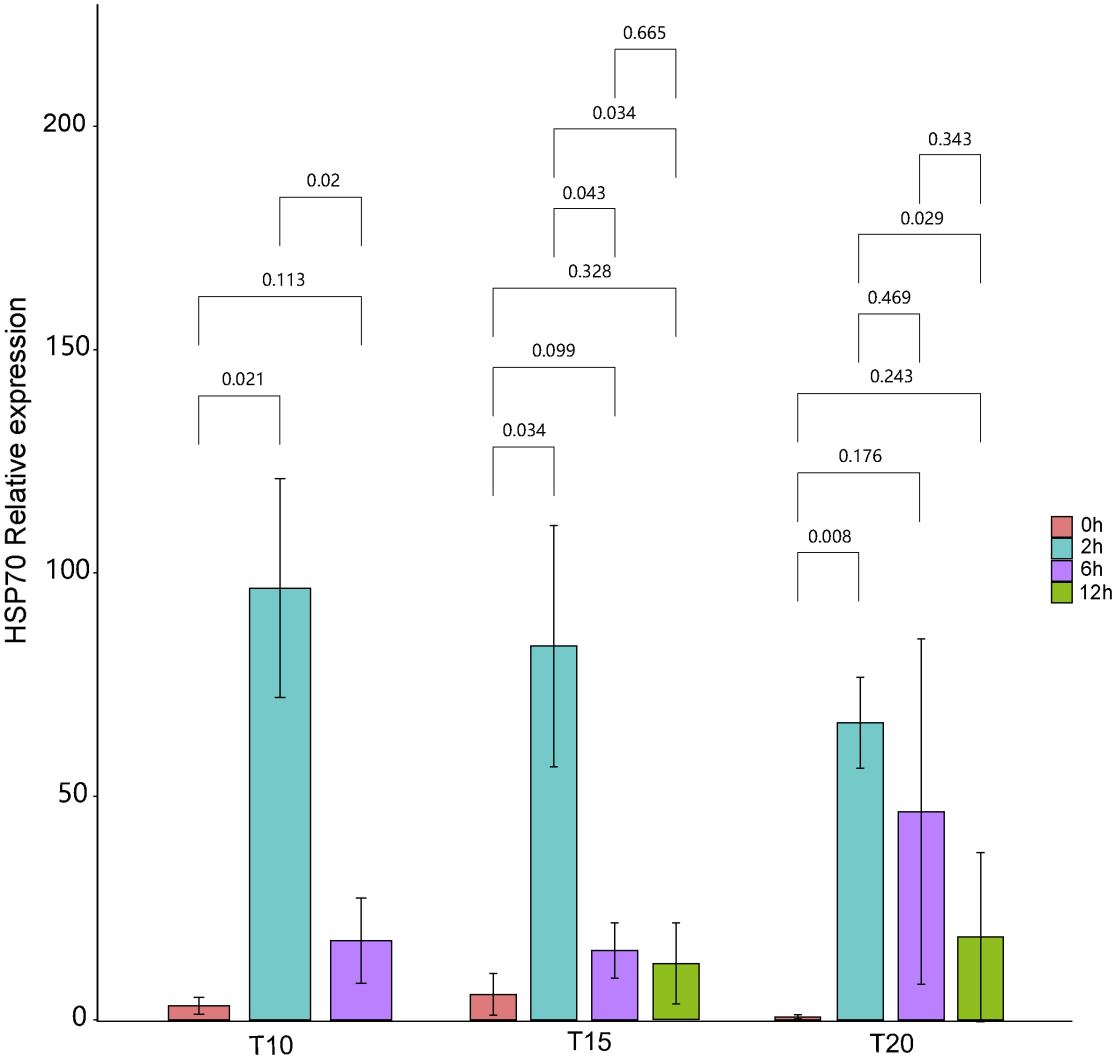
The relative expression of intestinal *HSP70* of heat stressed *O. stewarti* juveniles with different acclimation histories. T10, T15, and T20 indicate 10°C, 15°C, and 20°C acclimation temperature backgrounds of the experimental fish. The values 0–12 h represent the duration of the heat stress.

The expression of intestinal *HSP70* exhibited a “rising-declining” trend for fish from all of the acclimation treatment groups during the heat stress procedures (Figure 1). Before heat stress (0 h), the highest mean relative expression of intestinal *HSP70* (5.73 ± 4.66) in *O. stewarti* juveniles was recorded in the T15 treatment. Expression of intestinal *HSP70* rose rapidly after stress for 2 h, with the highest value (96.63 ± 24.44) in the T10 treatment. After stress for 6 h, the expression of intestinal *HSP70* decreased, with a maximum (46.61 ± 38.61) in the T20 treatment. After stress for 12 h, the mean relative expression of intestinal *HSP70* continued to decrease, with the lowest value (12.57 ± 9.03) in the T15 treatment.

### Intestinal Histological Variation Under Heat Stress

**Figure 2:**
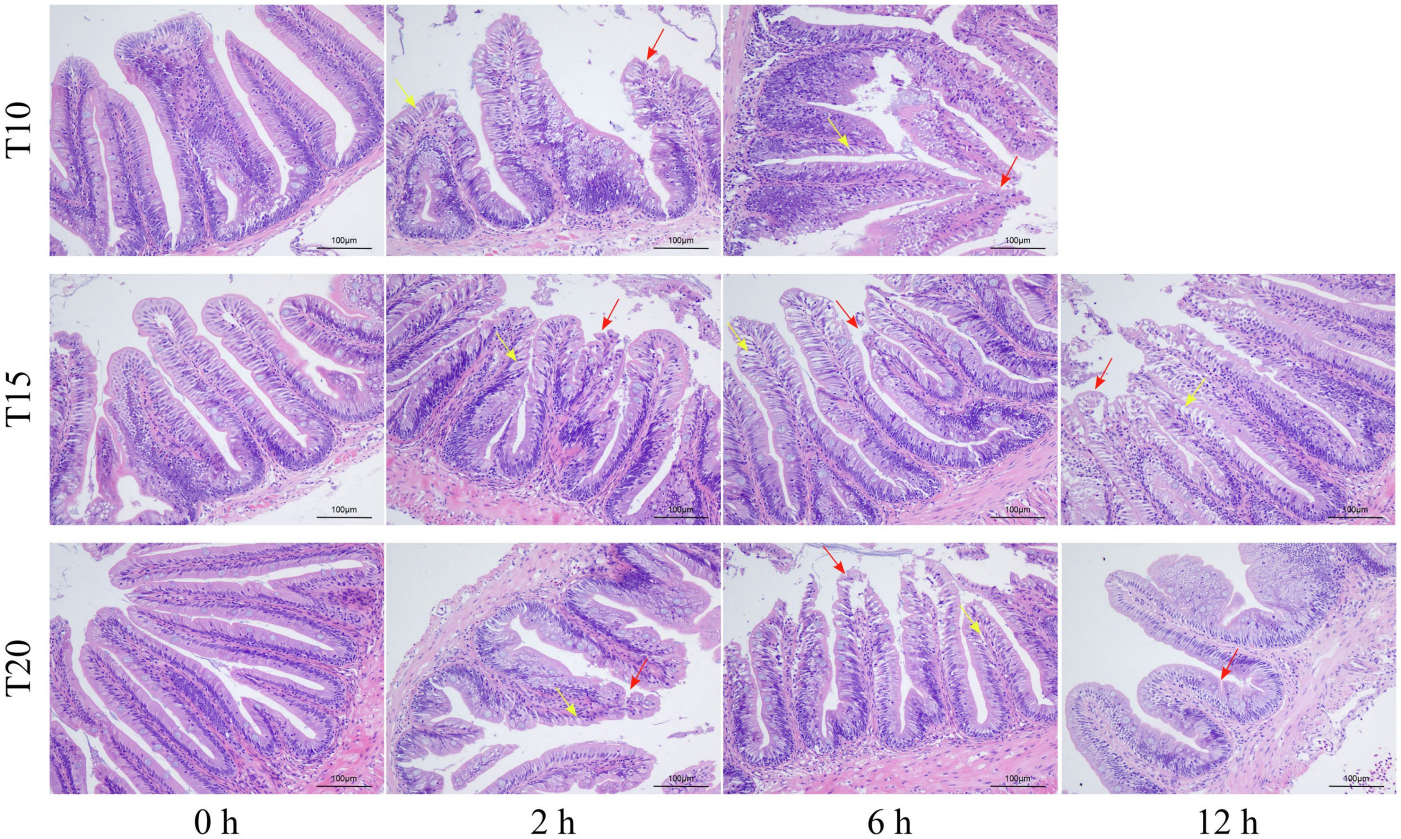
Sections of intestinal tissue of heat stressed *O. stewarti* juveniles with different acclimation histories. T10, T15, and T20 indicate 10°C, 15°C, and 20°C acclimation temperature backgrounds of the experimental fish. The values 0–12 h represent the duration of the heat stress. The red arrows show necrosis and shedding of intestinal villous epithelial cells; the yellow arrows show edema, swelling, and cytoplasmic light staining in the intestinal villous epithelial cells.

As shown by the intestinal histological analysis, heat stress caused varying degrees of damage to the intestines of experimental fish (Table 2; Figure 2). Before heat stress (0 h) there was no observable pathological changes in the intestines of *O. stewarti* juveniles for all treatments. After 2 h, diffuse infiltration of lymphocytes was observed in all treatments. After 6 h, intestinal villi epithelial cells were swollen, and cytoplasmic light staining was observed in all of the treatments, while in local intestinal villi epithelial cells necrosis and abscission were observed in the T10 and T20 groups. After 12 h, intestinal villi epithelial cells displayed swelling, and cytoplasmic light staining was observed in the T10 and T20 groups. The length of intestinal villi of all treatments consistently decreased with the prolonging of heat stress (Table 3).

**Table 2:** Intestinal histological variation of heat stressed *O. stewarti* juvenile with different acclimation histories.

Acclimation temperature (°C)	10	15	20
Heat stress for 0 h	The structure of intestinal mucosa was intact and no obvious pathological changes, such as inflammation, were found	The structure of intestinal mucosa was intact and no obvious pathological changes, such as inflammation, were found	The structure of intestinal mucosa was intact and no obvious pathological changes, such as inflammation, were found
Heat stress for 2 h	Some blood vessel lumen dilation can be seen in the muscular layer of intestinal tissue. Diffuse infiltration of lymphocytes was observed in the intestinal mucosa.	Diffuse infiltration of lymphocytes was observed in the intestinal mucosa.	Diffuse infiltration of lymphocytes was observed in the intestinal mucosa.
Heat stress for 6 h	Focal infiltration of lymphocytes can be seen in the mucosal layer of intestinal tissue. Local intestinal villi show epithelial cells necrosis and abscission. Some intestinal villi epithelial cells displayed edema, cell swelling, and cytoplasmic light staining.	Diffuse infiltration of lymphocytes was observed in the intestinal mucosa. Some intestinal villi epithelial cells displayed edema, cell swelling, and cytoplasmic light staining.	Local intestinal villi show epithelial cells necrosis and abscission. Some intestinal villi epithelial cells displayed edema, cell swelling, and cytoplasmic light staining.
Heat stress for 12 h		Local diffuse infiltration of lymphocytes can be seen in the mucosal layer of the tissue. Local intestinal villi show epithelial cells necrosis and abscission. Some intestinal villi epithelial cells display edema, cell swelling, and cytoplasmic light staining.	Local diffuse infiltration of lymphocytes can be seen in the mucosal layer of the tissue. Local intestinal villi show epithelial cells necrosis and abscission. Some intestinal villi epithelial cells display edema, cell swelling, and cytoplasmic light staining.

**Table 3:** Length change of intestinal villus of *O. stewarti* juveniles under different durations of heat stress.

Acclimation temperature (°C)	Length of intestinal villus (mm)			
	Heat stress for 0 h	Heat stress for 2 h	Heat stress for 6 h	Heat stress for 12 h
10	0.480 ± 0.038	0.387 ± 0.024	0.231 ± 0.031	-
15	0.393 ± 0.022	0.323 ± 0.011	0.274 ± 0.049	0.247 ± 0.028
20	0.466 ± 0.078	0.386 ± 0.016	0.312 ± 0.006	0.229 ± 0.022

Means followed by SD.

### Intestinal Microbiome Variation Under Heat Stress

**Figure 3:**
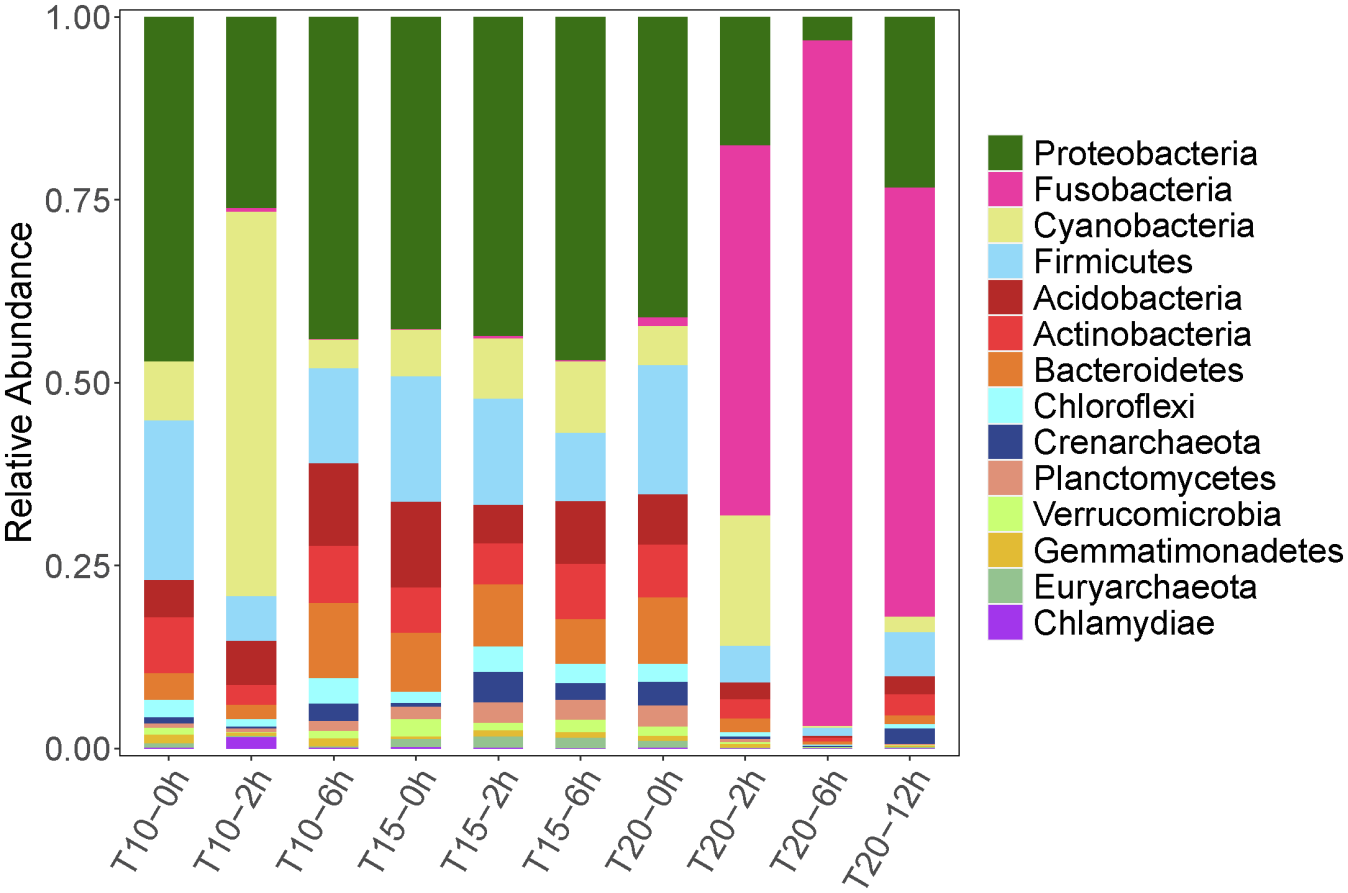
Relative abundance dynamics of the dominant intestinal bacterial phyla for juvenile *Oxygymnocypris stewarti* during heat stress. T10, T15, and T20 indicate 10°C, 15°C, and 20°C acclimation temperature background of the experimental fish; 0, 2, 6, and 12 h represent duration of the heat stress.

A total of 220,960 high-quality reads (average length = 420 bp) were generated from 10 intestinal samples, averaging 22,096 reads per sample. With a 97% sequence similarity cut off value, 26,535 OTUs were detected in total. The OTU number increased with the acclimation temperature, but heat stress induced a reduction in OTU number (Table 4). Among the microbial taxa, the dominant OTUs across all of the fish samples were classified into 14 phyla (Figure 3).

**Table 4:** Number of Operational Taxonomic Units (OTUs) detected in *O. stewarti* juveniles under different durations of heat stress.

Sample ID	Resampling sequence	OTUs	Singletons
T10-0h	10072	2,576	1709
T10-2h	10072	2,041	1452
T10-6h	10072	3,534	2301
T15-0h	10072	2,982	1927
T15-2h	10072	2,795	1617
T15-6h	10072	3,104	1903
T20-0h	10072	3,381	2187
T20-2h	10072	2,428	1802
T20-6h	10072	1,557	1350
T20-12h	10072	2,137	1578

T10, T15, and T20 indicate 10°*C, 15*°*C, and 20*°C acclimation temperature backgrounds of the experimental fish; 0, 2, 6, 12 h represent the duration of the heat stress.

Variation in intestinal microbiota of *O. stewarti* juveniles from different acclimation treatment groups was found during the heat stress procedure. Before the heat stress (0 h), the intestinal microbiota in the T10, T15, and T20 treatments were dominated by phyla Proteobacteria (45.9%, 38.8%, and 39.4%, respectively), Firmicutes (15.6%, 21.2%, and 17.0%, respectively), Acidobacteria (5.0%, 10.6%, and 6.5%, respectively), Cyanobacteria (7.9%, 5.8%, and 5.1%, respectively), Actinobacteria (7.5%, 5.7%, and 6.9%, respectively), and Bacteroidetes (3.5%, 7.3%, and 8.8%, respectively). After stress for 2 and 6 h, the intestinal microbial communities in T10 and T15 treatments were similar to those in the T10-0h and T15-0h treatments, except that phylum Cyanobacteria (51.3%) was most abundant in the T10-2h group. Interestingly, the dominant microbial phyla in T20-2h, T20-6h and T20-12h treatments were Fusobacteria (49.6%, 93.4%, and 57.7%, respectively), Proteobacteria (17.3%, 3.1%, and 23.1%, respectively), Cyanobacteria (17.5%, 0.2%, and 2.1%, respectively), and Firmicutes (4.9%, 1.1%, and 5.9%, respectively), results that were different from those in other treatments.

**Figure 4:**
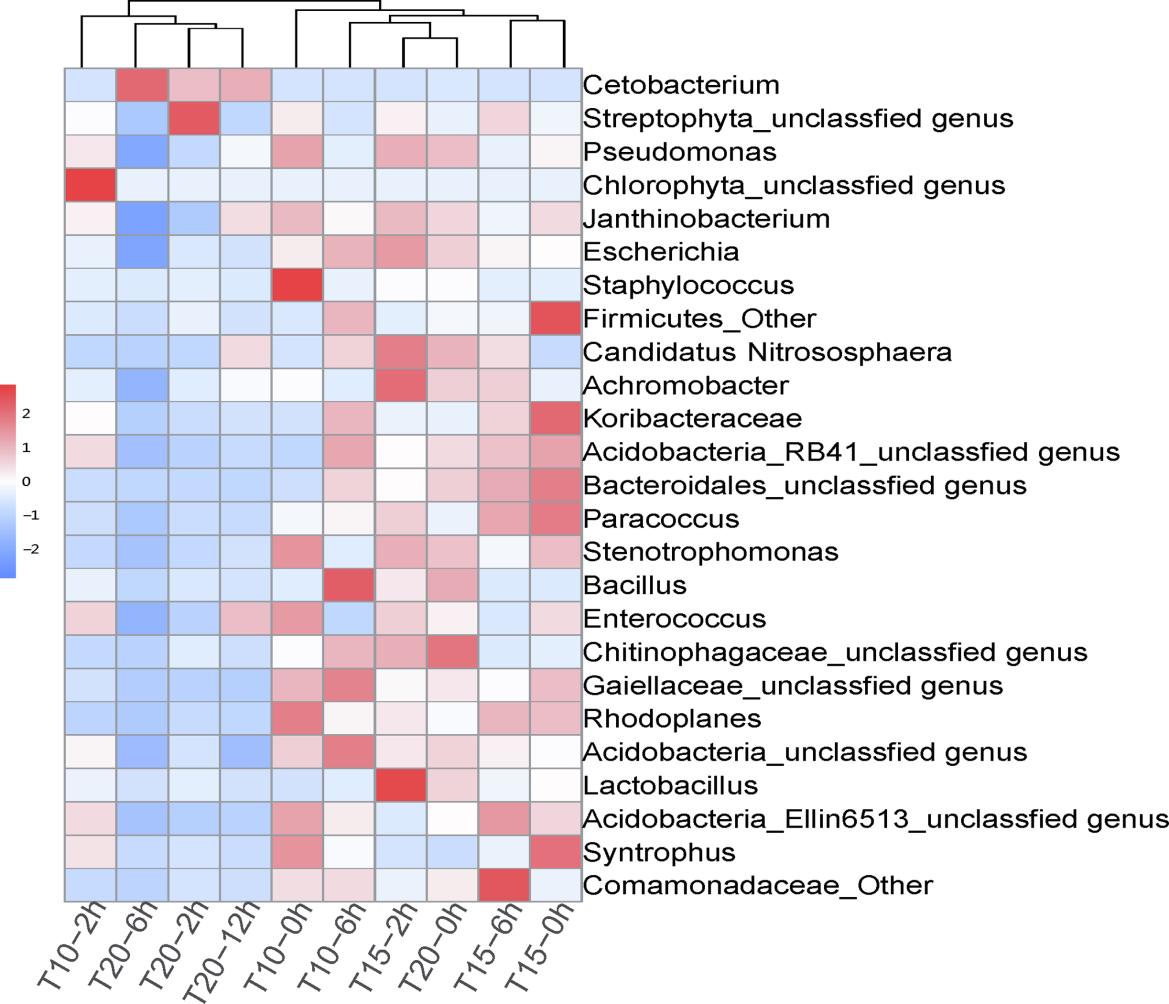
Correlation heatmap of dominant intestinal bacterial genera in juvenile *O. stewarti* samples during heat stress. T10, T15, and T20 indicate 10°C, 15°C, and 20°C acclimation temperature backgrounds of the experiment fish; 0, 2, 6, and 12 h represent the duration of the heat stress.

The correlation heatmap of dominant intestinal bacterial at the genus level showed significant changes among treatment groups, as T10-2h, T20-2h, T20-6h, and T20-12h clustered together, while T10-0h, T10-2h, T15-0h, T15-2h, T15-6h, and T20-0h clustered into another group (Figure 4). The dominant genus in T20-2h, T20-6h, T20-12h treatments was *Cetobacterium*, while the intestinal bacterial composition in other treatments was more diversified and included genera *Janthinobacterium*, *Escherichia*, *Staphylococcus*, *Candidatus* Nitrososphaera, *Achromobacter*, *Paracoccus*, *Stenotrophomonas*, and *Rhodoplanes*. Moreover, there were significant increases in the relative abundance of Chlorophyta unclassified genus in the T10-2h treatment and genera *Achromobacter* and *Lactobacillus* in T15-2h treatment as well as Streptophyta unclassified genus in the T20-2h treatment. With the extension of the heat stress duration, the intestinal bacterial composition tended to return to pre-stress conditions in the T10 and T15 treatments, while the recovery was limited in the T20 treatment.

**Figure 5:**
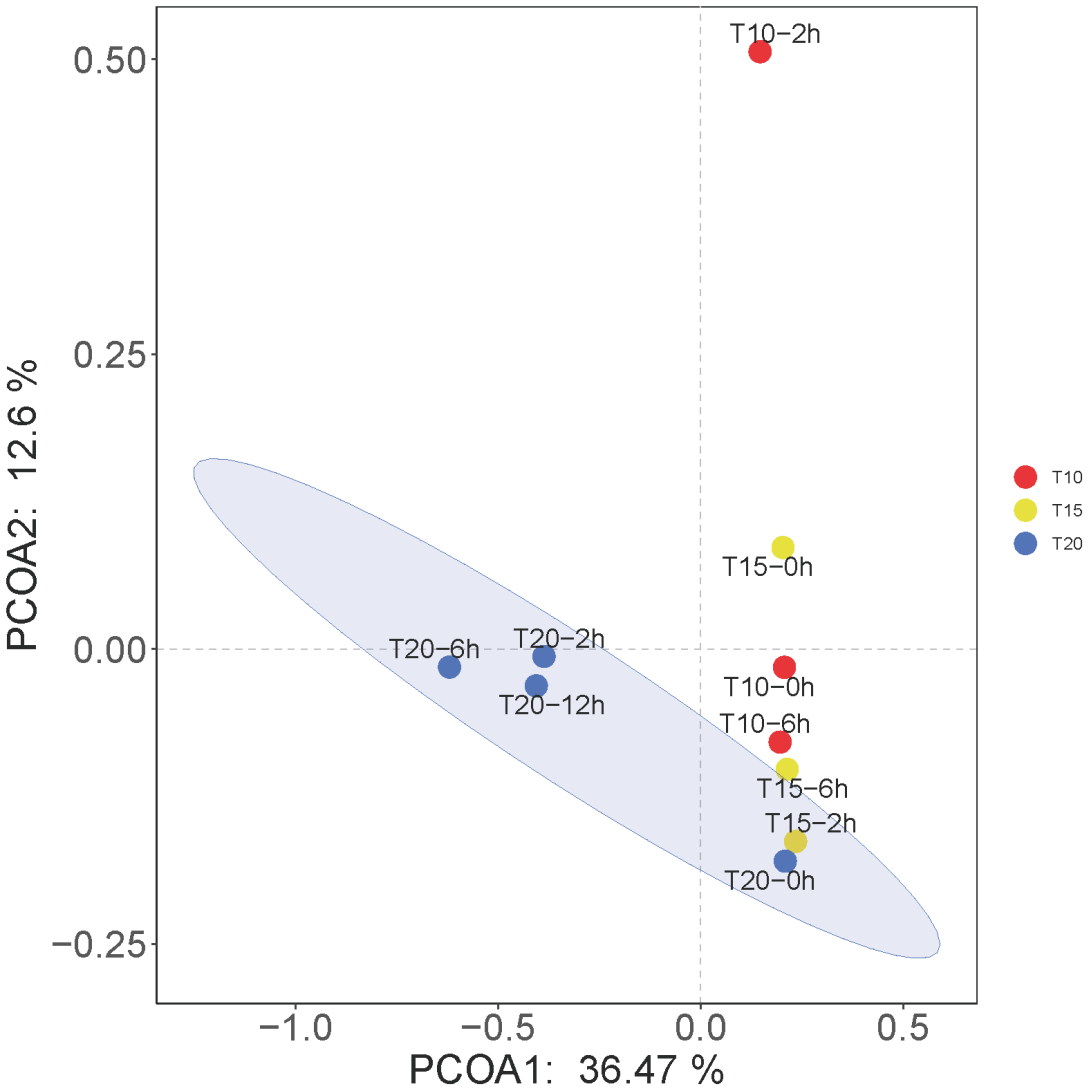
Principal coordinates analysis (PCoA) at the OTU levels of the intestinal microbiome of juvenile *O. stewarti*. T10, T15, and T20 indicate 10°C, 15°C, and 20°C acclimation temperature backgrounds of the experimental fish; 0–12 h represent the duration of the heat stress.

The results of PCoA showed that the microbial communities in T20-2h, T20-6h, and T20-12h treatments were similar and tended to cluster together, while T10-0h, T10-2h, T15-0h, T15-2h, T15-6h, and T20-0h clustered into another group, and T10-2h was clearly separated from the other treatments (Figure 5).

## Discussion

The crucial effects of temperature on organisms are widely recognized, but few studies have explored the compound effects of temperature acclimation and heat stress on fish. Such knowledge may be valuable for understanding the heat resistance of fish. In this study, we found significant effects of temperature acclimation on the growth, thermal tolerance, and intestinal heat stress response of juvenile *O. stewarti*. The treatments affected *HSP70* levels, intestinal histology, and the intestinal microbial communities of the fish.

### Effect of Temperature Acclimation on the Growth and Thermal Tolerance of *Oxygymnocypris stewarti*

In the present study the optimal growth temperature for the juvenile *O. stewarti* was about 15°C. Liu et al. (2018) had reported that a suitable growth temperature for *O. stewarti* juveniles was 14°C–17°C based on comparisons among five water temperature levels (5°C, 8°C, 11°C, 14°C, and 17°C), consistent with the results of our study. In our previous study, we also found that this temperature (15°C) was suitable for the growth of another juvenile Tibetan fish, *Schizopygopsis younghusbandi* (Zhu et al., 2019). Considering the temperature dependent production of *O. stewarti* fingerlings, the present results would be useful for its artificial reproduction and protection.

Previous studies had indicated that thermal tolerance was largely dependent on temperature acclimation (Chatterjee et al., 2004; Sarma et al., 2010; Fernando et al., 2016). The present study also showed that the thermal tolerance (i.e., *CT_Max_* and *LT_Max_*) of *O. stewarti* juveniles increased with the acclimation temperature, suggesting that temperature acclimation would be a good strategy to improve the heat stress tolerance of *O. stewarti*. However, the gain in thermal tolerance of *O. stewarti* and other Tibetan fishes through temperature acclimation was limited (Table 5) compared with those of subtropical and tropical fish species such as *Cyprinus carpio* (Chatterjee et al., 2004) and *Anabas testudineus* (Sarma et al., 2010). Therefore, it is reasonable to infer that Tibetan fishes are more vulnerable to climate warming.

**Table 5:** Thermal tolerance of *O. stewarti* and other reported Tibetan fishes.

Species	*T_acclimation_*	Heating rate	*CT_Max_*	*LT_Max_*	References
*Schizopygopsis younghusbandi*	10°C	1°C/30 min	30.98	31.76	Zhu et al., 2019
	15°C	1°C/30 min	31.72	32.14	
	20°C	1°C/30 min	32.01	32.31	
*Schizopygopsis younghusbandi*	12°C	1°C/h	32.3	-	Zeng et al., 2019
*Schizothorax oconnori*	12°C	1°C/h	32.4	-	
*Ptychobarbus dipogon*	12°C	1°C/h	30.2	-	
*Oxygymnocypris stewarti*	10°C	1°C/30 min	31.3	31.8	Present study
	15°C	1°C/30 min	32.1	32.6	
	20°C	1°C/30 min	32.3	32.5	

### Compound Effects of Temperature Acclimation and Heat Stress on Intestinal *HSP70* Levels

*HSP70* is an ideal indicator of heat stress, as it can enhance the resistance to heat. The expression of intestinal *HSP70* exhibited significant increases for *O. stewarti* in all of the acclimation treatment groups at a heat stress temperature of 30°C, but the magnitude of this induction was negatively correlated with the acclimation temperature. The common killifish *Fundulus heteroclitus* in populations from two different latitudes showed increased *HSP70-2* levels under heat stress, and the magnitude of this induction was greater in the high latitude population (Fangue et al., 2006). These studies further verified that fish from cold areas may be more susceptible to heat stress.

In addition, a decline of intestinal *HSP70* levels was found in *O. stewarti* from all acclimation treatment groups during 2–12 h of the heat stress experiment. Similar results were found in *Megalobrama amblycephala* (Ming et al., 2009). These results may be related to the gained heat tolerance of the heat stressed fish (Ming et al., 2009). However, the heart *HSP70* levels of broilers increased continuously under 48 h of persistent heat stress (40°C) conditions (Sun et al., 2007). The different variational characteristics of *HSP70* levels among different studies may be related to the differences in species, organs, and intensity of the heat stress.

It is worth noting that fish from T10 group were all dead after 6 h of heat stress. The possible reason may be limited heat resistance of fish from T10 group compared with fish from T15 and T20 group. Meanwhile, the heat stress temperature (30°C) was very close to the *CT_Max_* (31.3°C) of fish from T10 group, which indicated that death may occur if the heat stress lasts long enough.

### Response of Intestinal Histology Under Heat Stress

Most studies have shown adverse effects of heat stress on the intestinal integrity and function (Cronjé, 2005; Ortega and Szabó, 2021). An intact and functional intestinal epithelium is required for the intestine’s digestive, absorptive, and protective functions (Umar, 2010). The injury to the intestinal mucosa is often manifested as intestinal epithelial cell injury and apoptosis (Yang et al., 2015). High temperatures induced atrophy of the small intestinal mucosa and chorion in tilapia (Xie et al., 2008). Heat stress in chickens induced declines in the height, surface area, and volume of the small intestine epithelium (Uni et al., 2001) and led to acute enteritis (Quinteiro-Filho et al., 2010). Heat stress also reduced intestinal barrier integrity in pigs (Pearce et al., 2013). Meanwhile, intestinal integrity can be influenced by the duration and intensity of the exposure to heat stress (Ortega and Szabó, 2021). With the extension of heat stress duration, the intestinal injury to the *O. stewarti* juveniles became increasingly serious. The observed intestinal histological injuries included diffuse infiltration of lymphocytes, intestinal villi epithelial cell swelling, decrease of intestinal villi length, and cytoplasmic light staining. These signs of intestinal damage induced by heat stress may signal the weakening of nutritional metabolism and higher risk of infection.

### Compound Effects of Temperature Acclimation and Heat Stress on the Intestinal Microbiome

The present study demonstrated significant variation in the intestinal microbiome of *O. stewarti* juveniles under heat stress and different acclimation treatments. At the phylum level, the intestinal microbiota of temperature-acclimated *O. stewarti* juveniles were dominated by Proteobacteria. After heat stress, the dominant intestinal microbiota of *O. stewarti* juveniles in the T10 and T20 treatments were Cyanobacteria and Fusobacteria, respectively, while the intestinal microbial composition in the T15 treatment was stable compared with that before stress. This result was consistent with previous studies in which warm-temperature acclimation led to an increase in the relative abundance of Proteobacteria (Berg et al., 2016; Moghadam et al., 2018), and Fusobacteriales comprised the majority of the sequences in high temperature treated juvenile milkfish (*Chanos chanos*; Hassenrück et al., 2021). It is noteworthy that the intestinal microbial community showed a greater difference in the T20 treatment during stress and a weaker recovery time. A similar trend was also observed in milkfish (Hassenrück et al., 2021). At the genus level, genus *Cetobacterium* was dominant in the T20 treatment (2 h, 6 h, 12 h); this genus was reported to promote nutrient absorption of fish (Li et al., 2016). Six genera of bacteria (*Janthinobacterium*, *Staphylococcus*, *Achromobacter*, *Stenotrophomonas*, *Rhodoplanes*, and *Lactobacillus*) dominated the T10 and T15 treatments. Genus *Janthinobacterium* is common in temperate climates and cold climates, and genera *Staphylococcus* and *Achromobacter* are potential pathogens and could cause many forms of infection in fish and other animals (Foster, 1996), while genus *Lactobacillus* is marketed as a probiotic to protect the fish against pathogen infection (He et al., 2017). These results of pronounced variation in intestinal microbiota of heat stressed ectotherms indicates that such variation was closely related to the acclimation temperature. It has been reported that temperature also has important effects on the diversity and structure of the intestinal microbiota in ectotherms (Sepulveda and Moeller, 2020). Meanwhile, intestinal OTU number can respond to temperature change (Jaramillo and Castañeda, 2021). Here, we also found that both cold temperature acclimation and heat stress could induce obvious reduction in the intestinal OTU number in *O. stewarti* juveniles.

Due to various reasons, we did not examine the possibility of intestinal recovery of *O. stewarti* after heat stress. Future studies will need to address this important issue for a more comprehensive understanding of the heat resistance of Tibetan fish.

## Conclusion

Our study demonstrated that thermal tolerance depended on temperature acclimation, and the compound effects of temperature acclimation and heat stress on intestinal *HSP70* levels, intestinal histology, and the intestinal microbiome in juvenile *O. stewarti* were characterized. Overall, temperature acclimation improved the thermal tolerance and heat resistance of juvenile *O. stewarti*, but this fish species may be more vulnerable to climate warming compared with subtropical and tropical fishes. This study provides a theoretical and experimental basis for further research and protection of Tibetan fishes.

## Data Availability Statement

The datasets presented in this study can be found online: https://www.ncbi.nlm.nih.gov/bioproject/PRJNA798450.

## Ethics Statement

The animal study was reviewed and approved by the Institutional Animal Care and Use Committee of Yangtze River Fisheries Research Institute, Chinese Academy of Fishery Sciences.

## Author Contributions

TZ and XL conceived and designed the experiments and analyzed the data. TZ, XL, and XW performed the experiments. TZ, XL, and DY wrote the manuscript. All authors contributed to the article and approved the submitted version.

## Funding

This study was funded by the Central Public-interest Scientific Institution Basal Research Fund, CAFS (2020XT1302) and China Agriculture Research System of MOF and MARA (CARS-46).

## Conflict of Interest

The authors declare that the research was conducted in the absence of any commercial or financial relationships that could be construed as a potential conflict of interest.

## Publisher’s Note

All claims expressed in this article are solely those of the authors and do not necessarily represent those of their affiliated organizations, or those of the publisher, the editors and the reviewers. Any product that may be evaluated in this article, or claim that may be made by its manufacturer, is not guaranteed or endorsed by the publisher.

## References

[ref1] Alami-Durante H. Bergot P. Rouel M. Goldspink G. (2000). Effects of environmental temperature on the development of the myotomal white muscle in larval carp (*Cyprinus carpio* L.). J. Exp. Biol. 203, 3675–3688. doi: 10.1242/jeb.203.24.3675, PMID: 11076732

[ref2] Beitinger T. L. Bennett W. A. McCauley R. W. (2000). Temperature tolerances of north American freshwater fishes exposed to dynamic changes in temperature. Environ. Biol. Fish 58, 237–275. doi: 10.1023/A:1007676325825

[ref3] Berg M. Stenuit B. Ho J. Wang A. Parke C. Knight M.. (2016). Assembly of the *Caenorhabditis elegans* gut microbiota from diverse soil microbial environments. ISME J. 10, 1998–2009. doi: 10.1038/ismej.2015.253, PMID: 26800234 PMC5029150

[ref4] Bouchama A. Knochel J. P. (2002). Heat stroke. N. Engl. J. Med. 346, 1978–1988. doi: 10.1056/NEJMra011089 12075060

[ref5] Broderick N. A. Lemaitre B. (2012). Gut-associated microbes of *Drosophila melanogaster*. Gut Microbes 3, 307–321. doi: 10.4161/gmic.19896, PMID: 22572876 PMC3463489

[ref6] Bureau of Aquatic Products, Tibet, China (1995). Fishes and Fish Resources in Xizang, China. Beijing: China Agriculture Press.

[ref7] Cao Y. Liu Y. Dong Q. Wang T. Niu C. (2021). Alterations in the gut microbiome and metabolic profile in rats acclimated to high environmental temperature. Microb. Biotechnol. 15, 276–288. doi: 10.1111/1751-7915.13772, PMID: 33620148 PMC8719808

[ref8] Caporaso J. G. Kuczynski J. Stombaugh J. Bittinger K. Bushman F. D. Costello E. K.. (2010). QIIME allows analysis of high-throughput community sequencing data. Nat. Methods 7, 335–336. doi: 10.1038/nmeth.f.303, PMID: 20383131 PMC3156573

[ref9] Chatterjee N. Pal A. K. Manush S. M. Das T. Mukherjee S. C. (2004). Thermal tolerance and oxygen consumption of *Labeo rohita* and *Cyprinus carpio* early fingerlings acclimated to three different temperatures. J. Therm. Biol. 29, 265–270. doi: 10.1016/j.jtherbio.2004.05.001

[ref10] Chen M. Li B. Hou J. Pan Y. Zhaxi L. Wang W. (2017). Changes of microbial community after *Oxygymnocypris stewarti* death. Southwest China J. Agric. Sci. 30, 1233–1238. doi: 10.16213/j.cnki.scjas.2017.5.043

[ref11] Cronjé P. B. (2005). Heat stress in livestock—the role of the gut in its aetiology and apotential role for betaine in its alleviation. Recent Adv. Anim. Nutr. Aust. 15, 107–122.

[ref12] Desai A. S. Singh R. K. (2009). The effects of water temperature and ration size on growth and body composition of fry of common carp, *Cyprinus carpio*. J. Therm. Biol. 34, 276–280. doi: 10.1016/j.jtherbio.2009.03.005

[ref13] Dong L. Tong G. Yang X. Li L. Yan T. Ma K.. (2021). Genetic structure analysis of the cyprinid *Oxygymnocypris stewartii*. Aquacult. Fish Fish. 1, 66–74. doi: 10.1002/aff2.22

[ref14] Douglas A. E. (2018). Fundamentals of Microbiome Science: How Microbes Shape Animal Biology. Princeton, NJ: Princeton University Press.

[ref15] Edgar R. C. Haas B. J. Clemente J. C. Quince C. Knight R. (2011). UCHIME improves sensitivity and speed of chimera detection. Bioinformatics 27, 2194–2200. doi: 10.1093/bioinformatics/btr381, PMID: 21700674 PMC3150044

[ref16] Fangue N. A. Hofmeister M. Schulte P. M. (2006). Intraspecific variation in thermal tolerance and heat shock protein gene expression in common killifish, *Fundulus heteroclitus*. J. Exp. Biol. 209, 2859–2872. doi: 10.1242/jeb.02260, PMID: 16857869

[ref17] Fernando A. V. Lochmann S. E. Haukenes A. H. (2016). Critical thermal maxima of juvenile alligator gar (*Atractosteus spatula*, Lacépède, 1803) from three Mississippi-drainage populations acclimated to three temperatures. J. Appl. Ichthyol. 32, 701–705. doi: 10.1111/jai.13047

[ref18] Foster T. (1996). “Chapter 12: Staphylococcus,” in Medical Microbiology. 4th Edn. ed. Baron S. (Galveston (TX): University of Texas Medical Branch at Galveston). 21413252

[ref19] Hassenrück C. Reinwald H. Kunzmann A. Tiedemann I. Gärdes A. (2021). Effects of thermal stress on the gut microbiome of juvenile milkfish (*Chanos chanos*). Microorganisms 9:5. doi: 10.3390/microorganisms9010005, PMID: 33375015 PMC7822048

[ref20] He S. Ran C. Qin C. Li S. Zhang H. de Vos W. M.. (2017). Anti-infective effect of adhesive probiotic lactobacillus in fish is correlated with their spatial distribution in the intestinal tissue. Sci. Rep. 7:13195. doi: 10.1038/s41598-017-13466-1, PMID: 29038557 PMC5643340

[ref21] He Y. Wu X. Zhu Y. Li H. Li X. Yang D. (2014). Effect of rearing temperature on growth and thermal tolerance of *Schizothorax (Racoma) kozlovi* larvae and juveniles. J. Anim. Ecol. 46, 24–30. doi: 10.1016/j.jtherbio.2014.09.009, PMID: 25455937

[ref22] Hoffmann A. A. Sgrò C. M. (2011). Climate change and evolutionary adaptation. Nature 470, 479–485. doi: 10.1038/nature09670 21350480

[ref23] Hotchkiss R. Nunnally I. Lindquist S. Taulien J. Perdrizet G. Karl I. (1993). Hyperthermia protects mice against the lethal effects of endotoxin. Am. J. Phys. 265, R1447–R1457. doi: 10.1152/ajpregu.1993.265.6.r1447, PMID: 8285289

[ref24] Hoye B. J. Fenton A. (2018). Animal host: microbe interactions. J. Anim. Ecol. 87, 315–319. doi: 10.1111/1365-2656.12788 29442372

[ref25] Huo B. Xie C. Ma B. Yang X. Huang H. (2012). Age and growth of *Oxygymnocypris stewartii* (Cyprinidae: Schizothoracinae) in the Yarlung Tsangpo River, Tibet, China. Zool. Stud. 51, 185–194.

[ref26] Huo B. Xie C. Ma B. Yang X. Huang H. (2013). Reproductive biology of *Oxygymnocypris stewartii* in the Yarlung Zangbo River in Tibet, China. Environ. Biol. Fish 96, 481–493. doi: 10.1007/s10641-012-0031-4

[ref27] Huo B. Xie C. Madenjian C. P. Ma B. Yang X. Huang H. (2014). Feeding habits of an endemic fish, *Oxygymnocypris stewartii*, in the Yarlung Zangbo River in Tibet, China. Environ. Biol. Fish 97, 1279–1293. doi: 10.1007/s10641-013-0213-8

[ref28] Jaramillo A. Castañeda L. E. (2021). Gut microbiota of *Drosophila subobscura* contributes to its heat tolerance and is sensitive to transient thermal stress. Front. Microbiol. 12:654108. doi: 10.3389/fmicb.2021.654108, PMID: 34025608 PMC8137359

[ref29] Jia Y. Chen Y. (2009). Otolith microstructure of *Oxygymnocypris stewartii* (Cypriniformes, Cyprinidae, Schizothoracinae) in the Lhasa River in Tibet, China. Environ. Biol. Fish 86, 45–52. doi: 10.1007/s10641-008-9334-x

[ref30] Jia Y. Chen Y. (2011). Age structure and growth characteristics of the endemic fish *Oxygymnocypris stewartii* (Cypriniformes: Cyprinidae: Schizothoracinae) in the Yarlung Tsangpo River. Tibet. Zool. Stud. 50, 69–75.

[ref31] Kokou F. Sasson G. Nitzan T. Doron-Faigenboim A. Harpaz S. Cnaani A.. (2018). Host genetic selection for cold tolerance shapes microbiome composition and modulates its response to temperature. elife 7:e36398. doi: 10.7554/eLife.36398, PMID: 30454554 PMC6277203

[ref32] Leggett W. C. Carscadden J. E. (1978). Latitudinal variation in reproductive characteristics of American shad (*Alosa sapidissima*): evidence for population specific life history strategies in fish. J. Fish. Res. Board Can. 35, 1469–1478. doi: 10.1139/f78-230

[ref33] Li J. Hou J. Zhang P. Liu Y. Xia R. Ma X. (2016). Comparative study of intestinal microbial community structure in different species of carp in aquaponics system. South China Fish. Sci. 12, 42–50. doi: 10.3969/j.issn.2095-0780.2016.06.006

[ref34] Li H. Zhang N. Lin X. (2010). Spatio-temporal characteristics of Yarlung Zangbo River in Tibet. J. Hunan Univ. Nat. Sci. 38, 126–130. doi: 10.16366/j.cnki.1000-2367.2010.02.031

[ref35] Li J. Zhang H. Zhang X. Yang S. Yan T. Song Z. (2015). Molecular cloning and expression of two heat-shock protein genes (HSC70/HSP70) from Prenant's schizothoracin (*Schizothorax prenanti*). Fish Physiol. Biochem. 41, 573–585. doi: 10.1007/s10695-015-0030-4, PMID: 25690871

[ref36] Liu X. Chen B. (2000). Climatic warming in the Tibetan plateau during recent decades. Int. J. Climatol. 20, 1729–1742. doi: 10.1002/1097-0088(20001130)20:143.0.CO;2-Y

[ref37] Liu Y. Liu H. Liu S. Liu M. (2018). Effect of temperature on embryonic development and growth traits of *Oxygymnocypris stewartii* larvae and juvenile. Chin. J. Zool. 53, 910–923. doi: 10.13859/j.cjz.201806009

[ref38] Liu H. Xiao S. Wu N. Wang D. Liu Y. Zhou C.. (2019). The sequence and de novo assembly of *Oxygymnocypris stewartii* genome. Sci. Data 6:190009. doi: 10.1038/sdata.2019.9, PMID: 30720802 PMC6362891

[ref39] Luo Y. Wang Q. (2012). Effects of body mass and temperature on routine metabolic rate of juvenile largemouth bronze gudgeon *Coreius guichenoti*. J. Fish Biol. 80, 842–851. doi: 10.1111/j.1095-8649.2012.03229.x, PMID: 22471803

[ref40] Luo S. Zhang Q. De D. Jiang H. (2014). Analysis and evaluation on the nutrition composition in muscle of *Oxygymnocypris stewartii* in Lhasa River. J. Tibet Univ. 29:53. doi: 10.3969/j.issn.1005-5738.2014.01.002

[ref41] Ming J. Xie J. Liu B. He Y. Zhou Q. Pan L.. (2009). Cloning, sequence analysis of *HSP70* cDNA and effects of heat stress on its mRNA expression in *Megalobrama amblycephala*. J. Fish. Sci. China 16, 635–648. doi: 10.3321/j.issn:1005-8737.2009.05.001

[ref42] Moghadam N. N. Thorshauge P. M. Kristensen T. N. de Jonge N. Bahrndorff S. Kjeldal H.. (2018). Strong responses of *Drosophila melanogaster* microbiota to developmental temperature. Flying 12, 1–12. doi: 10.1080/19336934.2017.1394558, PMID: 29095113 PMC5927714

[ref43] Moltschaniwskyj N. A. Martınez P. (1998). Effect of temperature and food levels on the growth and condition of juvenile *Sepia elliptica* (Hoyle 1885): an experimental approach. J. Exp. Mar. Biol. Ecol. 229, 289–302. doi: 10.1016/S0022-0981(98)00058-6

[ref44] Ortega A. D. S. V. Szabó C. (2021). Adverse effects of heat stress on the intestinal integrity and function of pigs and the mitigation capacity of dietary antioxidants: a review. Animals 11:1135. doi: 10.3390/ani11041135, PMID: 33921090 PMC8071411

[ref45] Paladino F. V. Spotila J. R. Schubauer J. P. Kowalski K. T. (1980). The critical thermal maximum: a technique used to elucidate physiological stress and adaptation in fishes. Rev. Can. Biol. 39, 115–122.

[ref46] Pearce S. C. Mani V. Boddicker R. L. Johnson J. S. Weber T. E. Ross J. W.. (2013). Heat stress reduces intestinal barrier integrity and favors intestinal glucose transport in growing pigs. PLoS One 8:e70215. doi: 10.1371/journal.pone.0070215, PMID: 23936392 PMC3731365

[ref47] Quinteiro-Filho W. M. Ribeiro A. Ferraz-de-Paula V. Pinheiro M. L. Sakai M. Sá L. R. M.. (2010). Heat stress impairs performance parameters, induces intestinal injury, and decreases macrophage activity in broiler chickens. Poult. Sci. 89, 1905–1914. doi: 10.3382/ps.2010-00812, PMID: 20709975

[ref48] Sarma K. Pal A. K. Ayyappan S. Das T. Manush S. M. Debnath D.. (2010). Acclimation of *Anabas testudineus* (Bloch) to three test temperatures influences thermal tolerance and oxygen consumption. Fish Physiol. Biochem. 36, 85–90. doi: 10.1007/s10695-008-9293-3, PMID: 19082752

[ref49] Sepulveda J. Moeller A. H. (2020). The effects of temperature on animal gut microbiomes. Front. Microbiol. 11:384. doi: 10.3389/fmicb.2020.00384, PMID: 32210948 PMC7076155

[ref50] Sogard S. M. Spences M. L. (2004). Energy allocation in juvenile sablefish: effects of temperature, ration and body size. J. Fish Biol. 64, 726–738. doi: 10.1046/j.1095-8649.2004.00342.x

[ref51] Sun P. Liu Y. Zhao Y. Bao E. Wang Z. (2007). Relationship between heart damages and HSPs mRNA in persistent heat stressed broilers. Agric. Sci. China 6, 227–233. doi: 10.1016/S1671-2927(07)60039-X

[ref52] Umar S. (2010). Intestinal stem cells. Curr. Gastroenterol. Rep. 12, 340–348. doi: 10.1007/s11894-010-0130-3, PMID: 20683682 PMC2965634

[ref53] Underwood Z. E. Myrick C. A. Rogers K. B. (2012). Effect of acclimation temperature on the upper thermal tolerance of Colorado River cutthroat trout *Oncorhynchus clarkii pleuriticus*: thermal limits of a north American salmonid. J. Fish Biol. 80, 2420–2433. doi: 10.1111/j.1095-8649.2012.03287.x, PMID: 22650425

[ref54] Uni Z. Gal-Garber O. Geyra A. Sklan D. Yahav S. (2001). Changes in growth and function of chick small intestine epithelium due to early thermal conditioning. Poult. Sci. 80, 438–445. doi: 10.1093/ps/80.4.438, PMID: 11297282

[ref55] Wang Q. Garrity G. M. Tiedje J. M. Cole J. R. (2007). Naïve Bayesian classifier for rapid assignment of rRNA sequences into the new bacterial taxonomy. Appl. Environ. Microbiol. 73, 5261–5267. doi: 10.1128/AEM.00062-07, PMID: 17586664 PMC1950982

[ref56] Wen C. Li S. Wang J. Zhu Y. Zong X. Wang Y.. (2021). Heat stress alters the intestinal microbiota and metabolomic profiles in mice. Front. Microbiol. 12:706772. doi: 10.3389/fmicb.2021.706772, PMID: 34512584 PMC8430895

[ref57] Xie J. Wang G. Deng Y. Yu D. Wu L. Lu M. (2008). Effects of feeding thermostability enzyme (JM301) on the immunity and microvilli lying the gut of Tilapia. Fish. Moder. 35, 27–46.

[ref58] Xu J. Xie C. Shao J. Zhang H. (2011). Embryonic and larval development of *Oxygymnocypris stewartii* in the Yarlungzangbo River. J. Hydroecol. 32, 86–95. doi: 10.15928/j.1674-3075.2011.02.019

[ref59] Yang Y. Liu Z. Lu S. Li C. Hu P. Li Y.. (2015). Molecular cloning, expression and characterization of programmed cell death 10 from sheep (*Ovis aries*). Gene 558, 65–74. doi: 10.1016/j.gene.2014.12.040, PMID: 25541025

[ref60] Yi W. Cheng J. Wei Q. Pan R. Song S. He Y.. (2021). Effect of temperature stress on gut-brain axis in mice: regulation of intestinal microbiome and central NLRP3 inflammasomes. Sci. Total Environ. 772:144568. doi: 10.1016/j.scitotenv.2020.144568, PMID: 33770895

[ref61] You Q. Kang S. Wu Y. Yan Y. (2007). Climate change over the Yarlung Zangbo River basin during 1961-2005. J. Geogr. Sci. 17, 409–420. doi: 10.1007/s11442-007-0409-Y

[ref62] Zeng B. Zhang B. Mu Z. Liu H. Zhou J. Wang W. (2019). Tolerance of juveniles of three species native fishes in Tibet to water temperature. Fish. Sci. 38, 115–118. doi: 10.16378/j.cnki.1003-1111.2019.01.018

[ref63] Zhu T. Li X. Wu X. Yang D. (2019). Growth and thermal tolerance of a Tibet fish *Schizopygopsis younghusbandi* juveniles acclimated to three temperature levels. J. Appl. Ichthyol. 35, 1281–1285. doi: 10.1111/jai.13971

